# Effects of New Dietary Fiber from Japanese Apricot (*Prunus mume* Sieb. et Zucc.) on Gut Function and Intestinal Microflora in Adult Mice

**DOI:** 10.3390/ijms12042088

**Published:** 2011-03-25

**Authors:** Motoi Tamura, Yuriko Ohnishi, Tatsuya Kotani, Nobuki Gato

**Affiliations:** 1 National Food Research Institute, The National Agriculture and Food Research Organization, Tsukuba, Ibaraki, Japan; 2 Food Science Research Laboratory, Nakano BC Co. Ltd., Kainan, Wakayama, Japan; E-Mails: oonishi@nakano-group.co.jp (Y.O.); kotani@nakano-group.co.jp (T.K.); gato@nakano-group.co.jp (N.G.)

**Keywords:** ume, plasma lipids, fecal output, intestinal flora, dietary fiber

## Abstract

Much attention has been focused recently on functional foods. *Ume*, the Japanese name for the apricot of *Prunus mume* Sieb. et Zucc., is an example of a Japanese traditional functional food. There are, however, few reports on the effects of fiber from this fruit on bowel function. With this objective, we prepared *ume* fiber to test the hypothesis that it can change gut function and intestinal flora in mice. Mice were fed an *ume* fiber (UF) or cellulose (CF) diet (control) for 40 days. The fecal weight, fecal lipids, plasma lipids and cecal composition of the microflora were analyzed. The amount of feces was significantly greater in the UF group than in the CF group (p < 0.01). The fecal lipids content (% DW) of the feces sampled on the final day of the experiment were significantly greater in the UF group than in the CF group (p < 0.01). Plasma non-esterified fatty acids (NEFA) concentrations tended to be lower in the UF compared to the CF group (p = 0.058). Occupation ratios of *Bacteroides* and *Clostridium* cluster IV were significantly greater in the cecal flora of the UF group. Our results suggest that *ume* fiber possesses the fecal lipid excretion effects and feces bulking effects.

## 1. Introduction

Asian apricots have been used as daily foods and folk medicine in many Asian countries including Japan. The traditional drug *Bainiku-ekisu*, made from extracts of Japanese apricot (*Prunus mume* Sieb. et Zucc.), *ume* in Japanese, has been suggested to exert a powerful cardiovascular protective effect useful in the treatment of these diseases. It has also been reported that this drug markedly improves the fluidity of human blood. *Bainiku-ekisu* contains a novel compound, 1-[5-(2-formylfuryl)methyl] dihydrogen 2-hydroxypropane-1,2,3-tricarboxylate (mumefural), and a related compound, 5-hydroxymethyl-2-furfural (HMF) [1]. Mumefural was found to markedly improve blood fluidity in all human subjects tested [1]. Mumefural is one of a number of compounds produced during thermal processing of *ume* extracts; thus the processing itself seems to be important for developing the functionality of this fruit.

Dietary fiber is known to affect the gut environment [2]. Addition of fiber to the diet is recommended for treating constipation. However, it has been reported that there are differences in the effects of various dietary fibers on gastrointestinal transit time in humans. Pectin (soluble dietary fiber) was found not to significantly alter the mean stool pH, transit time or 24 h wet weight, while cellulose (insoluble dietary fiber) lowered the mean stool pH from 6.38 to 6.12, decreased mean stool transit time by 27% and increased mean stool wet weight by 57% [3]. While dietary pectin may not significantly affect the gastrointestinal transit time, it is nevertheless known to affect the gut environment. Animals given soluble polysaccharides had plasma enteroglucagon levels significantly higher than animals given insoluble cellulose [4]. A 10% pectin diet elicited a marked enlargement of the cecum, a drop in cecal pH and an increase in the volatile fatty acids (VFA) pool in rats [5]. Also in rats, a pectin-supplemented enteral diet was found to reduce the severity of methotrexate-induced enterocolitis [6].

*Ume* fruit contain both soluble and insoluble fiber. Recently, we developed a procedure to extract the dietary fiber from *P. mume.* While many functional characteristics have been reported for extracts of *ume*, as described above, there are few reports on the effects of the fiber extracts alone. In the present paper we have tested the hypothesis that fiber from this fruit changes gut function and intestinal flora in mice.

## 2. Results and Discussion

### 2.1. General Observations

No significant differences were observed between the UF and CF groups in final body weight (g) (UF 40.4 ± 0.8; CF 40.9 ± 0.4), in food consumption (g/day) (UF 4.4 ± 0.16; CF 4.4 ± 0.17), in visceral fat (g) (UF 2.74 ± 0.24; CF 2.90 ± 0.21) or in liver weight (g) (UF 1.66 ± 0.11; CF 1.63 ± 0.14).

### 2.2. Amount of Feces and Fecal Lipid Contents

**Figure 1.**
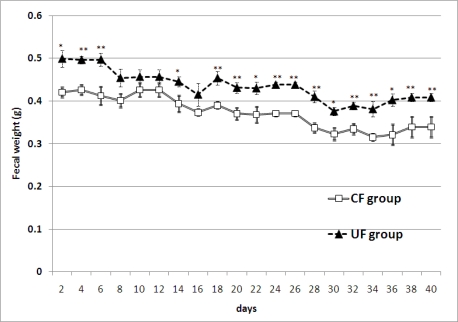
The amount of freeze-dried feces were significantly greater in the *ume* fiber (UF) group than in the cellulose (CF) group. Values are means ± SE (n = 7). The data were analyzed using *t*-test analysis. **Significantly different from the CF group (p < 0.01); *Significantly different from the CF group (p < 0.05).

**Figure 2.**
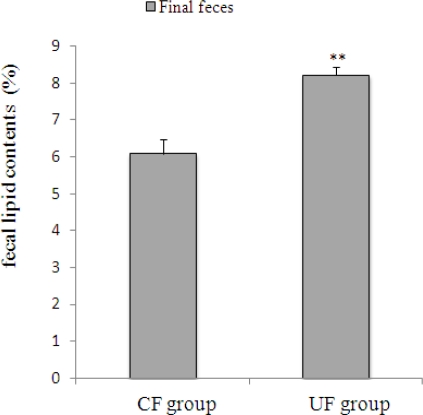
Fecal lipids contents (%) from the feces sampled on the final day of the experiment. The fecal lipids content (% DW) of the feces sampled on the final day of the experiment were significantly greater in the *ume* fiber (UF) group than in the cellulose (CF) group (p < 0.01) Values are means ± SE (n = 7). The data were analyzed using *t*-test analysis.

The UF diet significantly affected both the amount of feces and fecal lipid contents. The amount of feces (Figure 1) was significantly greater in the UF group than in the CF group. The fecal lipids content (% DW) of the feces sampled on the final day of the experiment were significantly greater in the UF group than in the CF group (p < 0.01) (Figure 2).

In the present study of the effects of fiber from the *ume* fruit on mice gut function and microflora, dry fecal weight was significantly increased in the UF compared to the CF group. It has been reported that diets containing wheat bran increased wet (by 67%) and dry (by 74%) fecal weight. However, diets containing soluble fiber in the form of pectin did not influence dry weight [7]. In our experiment, the CF diet did not contain soluble dietary fiber, while *ume* fiber contains both insoluble and soluble fibers. Insoluble dietary fiber plus soluble dietary fiber might effectively increase the amount of fecal output in the UF group. In our experiment, both UF and CF diets contained high fat (10%). Generally, incidence of tumors increases with increasing fat content in the diet [8]. It has been reported that tumorigenesis in rats was enhanced by increased fat content of the diet [9] while wheat bran was found to reduce tumor development in these animals [10]. In the latter report, the authors suggested that the effect of wheat bran could be due to a reduction of exposure of the colonic epithelium to carcinogens and promoters that are presumed to be necessary for tumor development. It has been reported that the dietary fiber inulin modulates parameters of colon cancer risks in human and animals colon cells. The mechanisms responsible possibly include reduction of exposure to risk factors and suppression of tumor cell survival. The dilution of carcinogenic factors to lower their contact with colon cells is considered to be one of the most important dietary strategies [11]. It has also been reported that increased incidence rate of colorectal tumors due to the intake of a soluble dietary fiber in chemically-induced rat carcinogenesis can be suppressed by partially substituting an insoluble fiber [12]. Due to its fecal bulking effect, and its mixture of insoluble and soluble polymers, *ume* fiber may thus be an interesting preventive dietary supplement against cancer of the colon. The soluble and insoluble dietary fiber seem to differently affect body weight in a long-term feeding. It has been reported that soluble *vs.* insoluble dietary fiber added to a high-fat, Western-style diet differently affected body weight [13]. In this report, a long-term study investigated potential protective effects of adding soluble guar fiber (10% w/w) *vs.* insoluble cereal fiber (10% w/w) to an isoenergetic and macronutrient matched high-fat diet in obesity-prone C57BL/6J mice. After 45 weeks, mice fed soluble *vs.* insoluble fiber showed significantly increased body weight (41.8 +/− 3.0 *vs.* 33.6 +/− 1.5 g, p = 0.03). In our study, there were no significant differences in final body weight between the UF and CF groups. It is expected that at least the soluble fraction of *ume* fiber shows stronger fermentation in comparison to cellulose. However, the experimental period was relatively short-term (40 days). So, further studies are needed to clarify the effects of *ume* fiber on body weight and obesity in long-term studies.

### 2.3. Plasma Total Cholesterol, Triglyceride, Phospholipids, NEFA and Plasma Glucose

**Figure 3.**
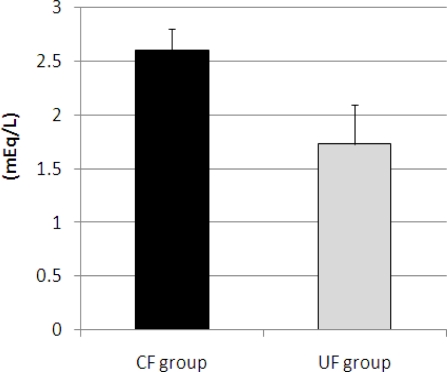
Plasma non-esterified fatty acids (NEFA) concentrations of mice in the *ume* fiber (UF) and cellulose (CF) groups. Values are means ± SE (n = 7). The data were analyzed using *t*-test analysis (p = 0.058).

At the end of the diet feeding period, the mice were anesthetized with diethylether and blood samples were taken from the abdominal aorta and placed in heparinized tubes. The plasma was separated from whole blood by centrifugation and used for analysis of plasma triglyceride, total cholesterol, phospholipids, NEFA and glucose. No significant differences in the plasma cholesterol (UF 205.2 ± 27.0 mg/dL; CF 263.3 ± 46.7 mg/dL), plasma triglyceride (UF 160.4 ± 10.7 mg/dL; CF 158.7 ± 23.1 mg/dL) or plasma phospholipids concentrations (UF 255.9 ± 21.8 mg/dL; CF 276.1 ± 24.0 mg/dL) were observed between the two groups. However, plasma NEFA concentrations tended to be lower in the UF than in the CF group (Figure 3) (p = 0.058). No significant differences between the two groups were observed for plasma glucose (UF 224.1 ± 24.2 mg/dL; CF 243.3 ± 30.7 mg/dL). In the present study, the increased fecal lipid content of the UF group compared to controls is likely to be related to the group’s lower plasma NEFA concentration. Pearson product-moment correlation coefficient between the plasma NEFA and fecal lipid content of two dietary groups were analyzed. Negative correlation (r = −0.632) was observed between the plasma NEFA and fecal lipid content of the two dietary groups.

### 2.4. Effects of Diet on Cecal Flora of Mice

**Figure 4.**
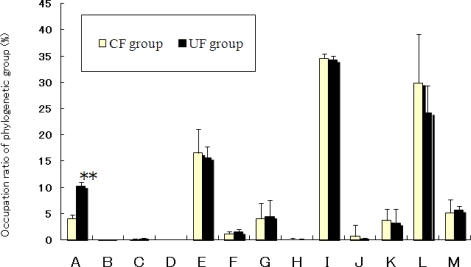
Composition of cecal intestinal microflora of mice in the *ume* fiber (UF) and cellulose (CF) groups. OTUs (operational taxonomic units), which correspond to either T-RFs (terminal restriction fragments) or T-RF clusters, detected by T-RFLP analysis. Values are means ± SE (n = 7). **Significantly different (p < 0.01) from the CF group. The data were analyzed using *t*-test analysis. The letters correspond to the following phylogenetic bacterial groups: **(A)** *Bacteroides, Clostridium* cluster IV (OTUs 370); **(B)** *Clostridium* cluster IV (OTUs 168, 749); **(C)** *Clostridium* cluster IX, *Megamonas* (OTUs 110); **(D)** *Clostridium* cluster XI (OTUs 338); **(E)** *Clostridium* subcluster XIVa (OTUs 106, 494, 505, 517, 754, 955, 990); **(F)** *Clostridium* cluster XI, *Clostridium* subcluster XIVa (OTUs 919); **(G)** *Clostridium* subcluster XIVa, *Enterobacteriales* (OTUs 940).; **(H)** *Clostridium* cluster XVIII (OTUs 423, 650); **(I)** *Bacteroides* (OTUs 469, 853); **(J)** *Bifidobacterium* (OTUs 124); **(K)** *Lactobacillales* (OTUs 332, 520, 657); **(L)** *Prevotella* (OTUs 137, 317); **(M)** Others.

The compositions of the phylogenetic groups of cecal flora differed between the two dietary groups (Figure 4). The occupation ratios of *Bacteroides* and *Clostridium* cluster IV were significantly greater in the UF than in the CF group (p < 0.01). It has been confirmed that intestinal microbiota predominantly consist of the members of approximately ten phylogenetic bacterial groups and that these bacterial groups can be distinguished by the T-RFLP system developed by Nagashima *et al.* [14,15]. There were significant differences in the composition of the intestinal flora between the two groups. It has been shown that consumption of apple pectin (7% in the diet) increases the population of butyrate- and beta-glucuronidase producing Clostridiales, and decreases the population of specific species within the Bacteroidetes group in the rat gut [16]. However, in our results, occupation ratios of *Bacteroides* and *Clostridium* cluster IV were significantly greater in the UF group. Different types of fiber may differently affect the intestinal flora of mice. Recently, much attention has been focused on the relation between intestinal flora and obesity. It has been reported that the amounts of *Bacteroides* are negatively correlated with fat pad mass, body mass and body-mass gain in rats [17]. Studies on human volunteers have revealed that obesity is associated with changes in the relative abundance of the two dominant bacterial divisions, the Bacteroidetes and the Firmicutes [18]. In rats, the *Bacteroides* group has been reported to affect the body mass and fat pad mass. In our experiment, despite the observed difference in bacterial populations, no such physical differences were observed between the two dietary groups. However, it is possible that the differences in bacterial composition observed were due to the differences of the fecal lipids content between two dietary groups. For our future research in this field, an investigation of the relationship between high fiber diets, including *ume* fiber, fat pad mass and the obesity phenotype and intestinal microflora, would be a most useful contribution.

## 3. Experimental Section

### 3.1. Production of Fiber from Fruit of Prunus mume

**Figure 5.**
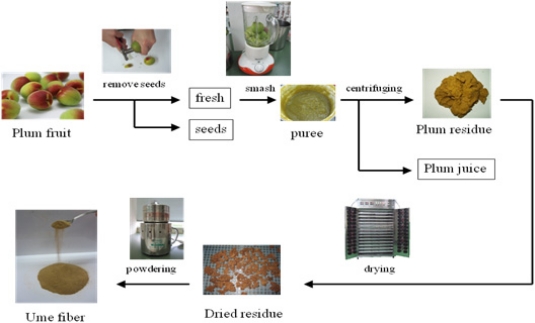
The method of manufacturing *ume* fiber.

The process of manufacturing *ume* fiber from *Prunus mume* is summarized in Figure 5.

Fiber from *ume* was prepared as follows. Whole *ume* (100 g), from which seeds were removed, were mashed to a puree. The puree (87.8 g) was then centrifuged at 8,000 rpm for 20 min (himac CR22G, HITACHI Co., Ltd., Tokyo, Japan). The sedimented material (23.5 g) was dried at 60 °C with a food drier (far-infrared radiation food drier vivi-9, Vianove Co., Ltd.) until the moisture content was less than 5%. The dried residue was ground down by mill (Wonder Crush/Mill WDL-1, Osaka Chemical Co., Ltd., Osaka, Japan) until the particle diameter of the powder was below 60 meshes. Finally, 3.8 g of *ume* fiber was obtained as a powder.

### 3.2. Treatment of Animals

Fourteen male Crj: CD-1 (ICR) mice (seven weeks old) were purchased from Charles River Japan, Inc. (Kanagawa, Japan). All mice were specific pathogen-free (SPF), and the animals were housed under conventional conditions in our laboratory. The mice were randomly divided into two groups of seven animals each and housed in suspended stainless-steel cages with wire mesh bottoms, in a room kept at 24 ± 0.5 °C, relative humidity 65%, with 12 h periods of light and dark. The mice were fed an AIN-93M diet for one week. The diet was then replaced with an *ume* fiber (UF) or cellulose (CF) diet (control) for 40 days. All mice were pair-fed. Table 1 presents the composition of each diet. The cellulose powder used in the CF diet was purchased from Oriental Yeast Co., Ltd.

The analysis of *ume* fiber was conducted by Japan Food Research Laboratories according to the manual of analytical methods for standard tables of food composition in Japan (Fifth Revised and Enlarged Edition). The nutritional components of *ume* fiber were as follows: moisture, 4.2%; protein, 13.1%; lipids, 3.1%; ash, 2.2%; carbohydrate, 15.2%, dietary fiber, 62.2% (soluble dietary fiber, 3.1%; insoluble dietary fiber, 59.1%). According to this information, we adjusted the composition of the UF or CF diets so that each had similar contents of fiber, protein, lipids and carbohydrate. Body weight, food consumption and amount of feces were measured during the experiment. Feces were dried by freeze dryer FD-1000 (Tokyo Rikakikai Co., Ltd., Tokyo, Japan) for 24 hr. Trap cooling temperature was −45 °C. Amounts of freeze-dried feces were also measured during the experiment.

### 3.3. Analysis of ume Fiber, Mice Feeding Conditions and Sampling

At the end of the diet feeding period, the mice were anesthetized with diethylether and blood samples were taken from the abdominal aorta and placed in heparinized tubes. The plasma was separated from whole blood by centrifugation and stored at −80 °C for later analysis of plasma triglyceride, total cholesterol, phospholipids, NEFA and glucose. The mice were then euthanized with diethylether. The liver and cecal contents were collected. Cecal contents were stored at −80 °C for analysis of intestinal microflora by T-RFLP. The liver samples and visceral fat were weighed. All procedures involving mice in this study were approved by the Animal Care Committee of the National Food Research Institute in Japan, in accordance with the “Guidelines for Animal Care and Experimentation” of the National Food Research Institute in Japan. The animal studies were reviewed and approved by the Animal Care and Use Committee of the National Food Research Institute, and the National Agriculture and Food Research Organization (NARO), Japan.

### 3.4. Measurement of Plasma Cholesterol, Triglyceride, Phospholipids, NEFA and Plasma Glucose

The following tests were performed with Wako kits obtained from Wako Pure Chemical Industries Ltd., Osaka, Japan. Total plasma cholesterol concentrations were measured using a cholesterol E-test Wako kit based on cholesterol oxidase [19]. Plasma triglyceride concentrations were measured using a triglyceride E-test Wako kit based on the glycerol-3-phosphate oxidase method [20]. Plasma phospholipid concentrations were measured using a phospholipid C-test Wako kit based on the choline oxidase method [21]. Plasma NEFA were measured using a NEFA C-test Wako kit based on the acyl-CoA synthase (ACS) and acyl-CoA oxidase (ACO) method. The plasma glucose concentrations were measured using a glucose C2-test Wako kit based on the mutarotase glucose oxidase method.

### 3.5. Fecal Lipid Extraction

Feces were dried by freeze dryer FD-1000 (Tokyo Rikakikai Co., Ltd., Tokyo, Japan) for 24 h. Trap cooling temperature was −45 °C. After drying, feces were milled by food mill TML17 (TESCOM Co., Ltd., Tokyo, Japan) for 30 s. Fecal lipid was extracted from the fecal powder by the Bligh and Dyer method [22].

### 3.6. DNA Extraction from Cecal Contents

DNA extractions from cecal contents were conducted according to the Matsuki’s method [23]. Cecal samples (20 mg) were washed three times by suspending them in 1.0 mL of phosphate-buffered saline and centrifuging each preparation at 14,000 g in order to remove possible PCR inhibitors. Following the third centrifugation the cecal pellets were resuspended in a solution containing 0.2 mL of phosphate-buffered saline and 250 μL extraction buffer (200 mM Tris-HCl, 80 mM EDTA; pH 9.0) and 50 μL 10% sodium dodecyl sulfate. Three hundred milligrams of glass beads (diameter 0.1 mm) and 500 μL of buffer-saturated phenol were added to the suspension, and the mixture was vortexed vigorously for 60 s using a Mini Bead-Beater (BioSpec Products Inc., Bartlesville, OK) at a power level of 4800 rpm. Following centrifugation at 14,000 g for 5 min, 400 μL of the supernatant was collected. Phenol-chloroform-isoamyl alcohol extractions were then performed, and 250 μL of the supernatant was subjected to isopropanol precipitation. Finally, the DNA was suspended in 1 mL Tris-EDTA buffer. The DNA preparation was adjusted to a final concentration of 10 μg/mL in TE and checked by 1.5% agarose gel electrophoresis.

### 3.7. PCR Conditions and Restriction Enzyme Digestion

The PCR mixture (25 μL) was composed of EX Taq buffer, 2 mM Mg^2+^ and each deoxynucleoside triphosphate at a concentration of 200 μM. The amount of cecal DNA was 10 ng. The primers used were 5′ HEX-labeled 516f (5′-TGCCAGCAGCCGCGGTA-3′) and 1510r (5′-GGTTACCTTGTTACGACTT-3′) at a concentration of 0.10 μM, template DNA and 0.625 U of TaKaRa EX Taq DNA polymerase (Takara Bio Inc., Otsu, Japan). This process was carried out using the Dice PCR System (Takara Bio Inc.). Amplification was performed with one cycle at 95 °C for 15 min, followed by 30 cycles at 95 °C for 30 s, 50 °C for 30 s, 72 °C for 1 min, and finally one cycle at 72 °C for 10 min. The amplification products were subjected to gel electrophoresis in 1.5% agarose followed by ethidium bromide staining. The PCR products were purified using QIAquick spin columns (Qiagen KK, Tokyo, Japan) according to the manufacturer’s instructions. The purified DNA was treated with 2 U of Bsl*I* (New England Biolabs) for 3 h, at 55 °C [14].

### 3.8. T-RFLP Analysis

The fluorescently labeled T-RFs were analyzed by electrophoresis on an ABI PRISM 310 Genetic Analyzer automated sequence analyzer (Applied Biosystems) in GeneScan mode. The restriction enzyme digestion mixture (2 μL) was mixed with 0.5 μL of MapMarker 1000 size standard (BioVentures, Inc.) and 12 μL of deionized formamide. The mixture was denatured at 96 °C for 2 min and immediately chilled on ice. The injection time was 30 s for analysis of T-RFs from the digestion with Bsl*I*. The run time was 40 min. The lengths and peak areas of T-RFs were determined with the GeneMapper software. From the predominant operational taxonomic units (OTUs, which correspond to either T-RFs or T-RF clusters) that were detected in the T-RFLP profiles, phylogenetic groups of intestinal flora were identified [14,15].

### 3.9. Statistics

The data are expressed as the mean ± standard error (SE). All data were analyzed using the Sigma Plot 11 (Systat Software, Inc., CA, USA). Pearson product-moment correlation coefficient between the plasma NEFA and fecal lipid content of two dietary groups were analyzed. The remaining data were analyzed using *t*-test analysis. Statistical significance was reached with a P value of less than 0.05.

## 4. Conclusions

In conclusion, fecal output was significantly increased in the UF group compared to the CF group. Both fecal weight and fecal lipid concentration on the final day were significantly greater in the UF than in the CF groups. There were significant differences in the composition of the intestinal flora between two groups. Occupation ratios of *Bacteroides* and *Clostridium* cluster IV were significantly greater in the UF group. The characteristics described for *ume* fiber, including its feces bulking effects, suggest that this new fiber possesses the fecal lipid excretion effects and feces bulking effects.

## Figures and Tables

**Table 1.** Composition of the experimental diet.

**Ingredient (g/kg diet)**	**AIN-93M**	**UF diet**	**CF diet**
Corn starch	465.692	388.246	405.686
Casein	140	129.52	140
α-Corn starch	155	155	155
Sucrose	100	100	100
Rice bran oil	-	97.52	100
Soy bean oil	40	-	-
Cellulose	50	-	50
Ume fiber	-	80.4	-
Mineral mix (AIN-93M-Mix)	35	35	35
Vitamin mix (AIN-93-Mix)	10	10	10
L-Cystine	1.8	1.8	1.8
Choline Bitartrate	2.5	2.5	2.5
Tert-butylhydroquinone	0.008	0.014	0.014
